# Getting inside acupuncture trials - Exploring intervention theory and rationale

**DOI:** 10.1186/1472-6882-11-22

**Published:** 2011-03-17

**Authors:** Sarah Price, Andrew F Long, Mary Godfrey, Kate J Thomas

**Affiliations:** 1 School of Healthcare, University of Leeds, Baines Wing, Leeds LS2 9DU, UK; 2 Leeds Institute of Health Sciences, University of Leeds, 101 Clarendon Road, Leeds LS2 9LJ, UK

## Abstract

**Background:**

Acupuncture can be described as a complex intervention. In reports of clinical trials the mechanism of acupuncture (that is, the process by which change is effected) is often left unstated or not known. This is problematic in assisting understanding of how acupuncture might work and in drawing together evidence on the potential benefits of acupuncture. Our aim was to aid the identification of the assumed mechanisms underlying the acupuncture interventions in clinical trials by developing an analytical framework to differentiate two contrasting approaches to acupuncture (traditional acupuncture and Western medical acupuncture).

**Methods:**

Based on the principles of realist review, an analytical framework to differentiate these two contrasting approaches was developed. In order to see how useful the framework was in uncovering the theoretical rationale, it was applied to a set of trials of acupuncture for fatigue and vasomotor symptoms, identified from a wider literature review of acupuncture and early stage breast cancer.

**Results:**

When examined for the degree to which a study demonstrated adherence to a theoretical model, two of the fourteen selected studies could be considered TA, five MA, with the remaining seven not fitting into any recognisable model. When examined by symptom, five of the nine vasomotor studies, all from one group of researchers, are arguably in the MA category, and two a TA model; in contrast, none of the five fatigue studies could be classed as either MA or TA and all studies had a weak rationale for the chosen treatment for fatigue.

**Conclusion:**

Our application of the framework to the selected studies suggests that it is a useful tool to help uncover the therapeutic rationale of acupuncture interventions in clinical trials, for distinguishing between TA and MA approaches and for exploring issues of model validity. English language acupuncture trials frequently fail to report enough detail relating to the intervention. We advocate using this framework to aid reporting, along with further testing and refinement of the framework.

## Background

Acupuncture explored in clinical trials is often reported as if it were a clearly defined intervention, which can be taken at face value. In contrast, the theory and practice of acupuncture suggests acupuncture is more accurately described as a complex intervention, with multiple variations in its form of practice and considerable variation in the knowledge and skills of its practitioners and in the theory of healing underpinning them [1]. Moreover, unless researched within the context of placebo controlled trial, the specific and non-specific effects of acupuncture are difficult to pick apart [2]. For example, in attempting to clarify the nature and effects of complex interventions such as acupuncture, Paterson and Dieppe [3] usefully differentiate characteristic from incidental (placebo) effects; characteristic effects of an intervention are defined as being 'theoretically derived, unique to a specific treatment, and believed to be causally responsible for the outcome' (p1202). What is defined as a characteristic and incidental effect will depend on the underlying therapeutic framework that informs the work, for example in the context of acupuncture, a biomedical or Chinese medicine framework. As Paterson and Dieppe report, the biomedical explanation of the symptom (diagnosis) may not drive the rationale for the acupuncture treatment administered in the trial if using a Chinese medicine framework.

This potentially poses considerable challenges in building up an evidence base on the effectiveness of acupuncture. Acupuncture trials produce variable and sometimes inconclusive results [4-6]. With possibly limited detail on the nature of the acupuncture studied within the trial and the underlying therapeutic framework, the results may be difficult to apply to, and to reproduce within, practice. In particular, the mechanism of acupuncture (the process by which the desired change is effected) is often unclear, or may vary depending on the theory driving it, raising questions over the validity of the acupuncture intervention itself [7].

Previous work to address this issue is most notable in the published guidelines on standards for reporting acupuncture trials (STRICTA), first published in 2001 [8] and revised in 2010 [9]. These are intended to improve the completeness and transparency of reporting with a view to enable better interpretation and to allow for replication. Our own interest, and the focus of this paper, lies on the first item within the STRICTA checklist, 'acupuncture rationale.' This draws attention in particular to the need for information on the style of acupuncture and the reasoning for the provided treatment and treatment procedures [9].

Another set of literature raises the connected but wider notion of model validity [10-12]. This draws attention to the need for research to 'fit the paradigm' [10] of the whole system of the particular complementary and alternative therapy under investigation. It asks questions about whether or not the research design takes heed of its underlying philosophy and theory of healing, and thus is assessed within its own explanatory model. This relates *inter alia *to the practice context (is the studied intervention consistent with current practice?), the credibility of the intervention (is it credible that an intervention based on this explanatory model could lead to the intended effects?), treatment procedures (are these congruent with the underlying healing system?)[12,13], and understanding of health-related changes arising from the intervention (for example, are the outcomes to be assessed sufficiently broad to encompass the effects of the therapy?)[14-16].

In her recent editorial, Cassidy [11] redraws attention to the complexity of acupuncture. This relates to its multiple styles, approach to needling, attention to the patient within the treatment session and reassessment at a subsequent session. Later, she asks, but 'what do we mean by acupuncture?'[12], and in contrast to biomedicine, what is its explanatory model? The revised Medical Research Council (MRC) guidelines for the conduct and reporting of trials of complex interventions also emphasise the early need for a theoretical understanding of how the intervention might bring about change [17]. Ensuring model validity thus enables clarity in assessing whether the intervention has had the expected effect in a clinical trial.

## Aim

Our aim was to aid the identification of the explanatory model underlying acupuncture interventions and the assumed mechanisms within clinical trials by developing an analytical framework to differentiate two contrasting approaches to acupuncture (traditional acupuncture and Western medical acupuncture).

## Methods

### Uncovering the Underlying Theory

The development of the analytical framework was grounded within the principles of realist review [18]. Interest lies in making explicit how and why an intervention may result in particular outcomes. Realist review applies the principles of realist evaluation to the process of research synthesis. Its first step is to uncover or identify the essential or implicit theory or theories that underlie an intervention, that is, how the intervention is thought or meant to work and its expected impacts. In the current context, this is akin to clarifying the underlying therapeutic rationale that guides and lies implicit within the acupuncture intervention [19].

### Developing the Analytical Framework

Within the context of Western professional acupuncture practice and research, two contrasting approaches to acupuncture are evident: traditional acupuncture (TA) grounded within Chinese or Japanese traditional medicine theory, and medical acupuncture (MA) based on a biomedical approach. Both are also differentiated in terms of the nature and length of education and training required for their practice [20].

Five key components of potential difference can be identified: 1) theory (for aetiology, diagnosis and treatment); 2) the rationale offered for how the treatment is expected to work; 3) the practice of the intervention; 4) the implied or assumed causal pathway for change; 5) the desired outcome of treatment. While these five components are interrelated, the underlying theory of acupuncture underpins them all. For example, the theory of Chinese medicine talks in terms of the balance of Qi, embracing how to diagnose, and how to formulate a treatment strategy. The practice should embody the theory; for instance, as Chinese medicine holds to the idea of holism, then its practice must reflect the understanding that mind, body and spirit are integral.

To develop the framework reported here, the educational content and guidelines of two UK professional bodies for acupuncture were examined to identify the core features of each approach. For TA, material from the British Acupuncture Council was examined; their members predominantly subscribe to a 'traditional acupuncture' perspective drawing on Chinese medicine theory. For MA, material from the British Medical Acupuncture Society was explored; their members are medically trained and thus can be characterised as subscribing to a 'medical acupuncture' approach. This material was supplemented by *a priori *knowledge of one of the authors (SP) [21], a practising acupuncturist-researcher with 25 years practice experience.

Based on these materials one can infer that acupuncturists that practise TA use Chinese and Japanese traditional medicine theory, which for the purposes of this paper we will shorten to Chinese Medicine (CM). Its mechanism, that is, the way in which change comes into being, is essentially individualistic, iterative and interactive with degrees of uncertainty at each stage of treatment, that is, the causal pathway is non-linear [22]. In contrast, MA uses 'neuro-physiological principles' based on evidence that acupuncture alters brain function through stimulating nerve pathways [23]. This is suggestive of a mechanism that has a linear pathway from acupuncture to symptom.

The resulting framework, presented in Table 1, displays a data extraction tool for categorising the acupuncture intervention provided. This framework makes explicit the differentiating features of a TA or MA model. Features of both systems that were about values rather than principles of theory were not included. Two points are noteworthy. Firstly, the heading '*aetiology of the symptom *as integral to the treatment' is considered exclusively a TA feature; how a symptom starts provides diagnostic information for this type of acupuncture. Secondly, the heading '*responsiveness' *is important as patient responses and changes provide new diagnostic information that may modify the treatment approach (iterative treatment). This is a feature that orientates the TA model to the individual over and above the symptom. In contrast, the MA model focus stays fixed on the symptom.

**Table 1:** Differentiation of theory, rationale, practice and outcome for two models of acupuncture (see text for more detailed definitions)

TA	Component	MA
Approach is orientated to the whole person based on an understanding that mind, body and spirit are integral	**Theory**	Approach is focused on the symptom

Aetiology of the main complaint: a detailed history of the main complaint is integral to the diagnosis	**Theory (Aetiology)**	Aetiology of the symptom is prognostic only and does not affect process of treatment.

▪ Differential Diagnosis: summary of the core curriculum theory of TCM encompassed by Bian Zheng or pattern differentiation	**Theory (Diagnosis)**	▪ Symptom based diagnosis
▪ Diagnostic Methods: principles of looking, listening, asking, touching (tongue and pulse) are part of differential diagnosis		▪ Diagnostic Methods: concerned with nature and location of symptom

▪ Treatment Principles: understanding of main complaint in terms of context, nature and location including concepts of *xu *and *shi*, and *ben *and *biao *(specific CM terminology)	**Theory (Treatment)**	▪ Treatment Principles: the symptom and its underlying biomedical mechanism have a linear path
▪ Responsiveness: diagnosis and treatment change in response to patient reported changes		▪ Constant: treatment approach stays focussed on the principle of affecting change in the biomedical mechanism of the symptom

▪ Mechanism of change is dependent on affecting the balance of Qi, Yin and Yang and other defined substances of the person as decided by the differential diagnosis	**Rationale**	▪ Mechanism of change is based in biomedical science and is linked to a direct expected action of acupuncture
▪ Acupuncture works by affecting change in the balance of Qi, Yin and Yang and other imbalances as defined by the differential diagnosis.		▪ Biomedical mechanism of acupuncture whether hypothesised or tested is t linked to the symptom

An iterative, individualistic and interactive approach - At each session the practitioner uses the principles of looking, listening, asking, touching (tongue and pulse) to make a differential diagnosis and to formulate a new treatment.	**Practice**	At each session, the selection for the formulae of points remains fixed on the biomedical mechanism of the symptom.

Non-linear, with degrees of uncertainty at each stage of treatment	**Possible Causal Pathway**	Linear, from acupuncture needling to symptom

Desired changes in the balance of Qi, Yin and Yang and other imbalances as defined by the differential diagnosis which will manifest in various aspects of the person in mind and body, in addition to any specific symptom changes	**Outcome**	A change in dimensions of the symptom, for example, hot flushes, a change in the intensity and frequency.

### Methods for Applying the Framework

This framework was then applied to a set of clinical trials to explore its usefulness in uncovering the theory behind the treatment approach and choice of acupuncture points and thus its ability to expose the stated or assumed underlying causal process of change. In order to do this we applied the conceptual framework to one body of research - published trials of acupuncture for fatigue or vasomotor symptoms. The vast majority of acupuncture research has been conducted with pain for which the suggested causal pathway is simple - a release of beta-endorphins [24]. Choosing two symptoms that are commonly experienced and researched but that might represent greater complexity in terms of mechanism allows a more in-depth exploration of the value of the framework.

The studies were selected from a separate, wider systematic literature review being undertaken within a PhD study of acupuncture and early stage breast cancer. The inclusion criteria for this separate systematic review were:

• *Study type*: randomised and non-randomised studies, with a comparison/control group; uncontrolled studies published in key journals and/or cited by subsequent studies;

• *Outcome measures*: validated measures for either fatigue or vasomotor symptoms;

• *Acupuncture*: to include needling;

Only studies reported in the English language were included, all other studies were excluded, including where acupuncture was used as part of a pregnancy or fertility trial, or acupuncture took the form of auricular acupuncture, laser therapy, acupressure or TENS or used healthy subjects. We systematically searched nine databases from 1986 to 2008: (1) the major biomedical databases of Medline, Embase, CINAHL, PsychInfo and the Cochrane database of systematic reviews; and (2) the specialist databases of AMED, CISCOM, Acubriefs, and Cochrane Complementary Medicine Field Registry. A hand search of English language Chinese medicine journals (Journal of Chinese Medicine, European Journal of Oriental Medicine) was also undertaken. Search terms were 'acupuncture' or 'acupuncture therapy' combined with the above symptoms.

This resulted in a set of fourteen studies. Following the realist evaluation approach, interest lay in uncovering implicit theory that the studies' authors might suggest about how the intervention was thought or meant to work and its expected effects. Focus thus lay on examining the authors' statements about aims, hypotheses and underlying rationale which might be evident within either the introduction or discussion sections of the article. The framework was applied systematically to the selected papers, firstly to assess the degree to which information could be identified demonstrating adherence to a particular theoretical model and secondly to explore any differences in model adherence by symptom.

## Results

### Applying the Framework

#### Adherence to a Theoretical Model

The results suggest that studies could fit into one of three models or categories, TA, MA, and 'unclassifiable', that is, where there is insufficient information to identify a fit to either model. Table 2 provides detail for all fourteen studies including the rationale for the categorisation of each study.

**Table 2:** All included studies

Authors	Outcome Measure	Authors' description of acupuncture treatment	N	**Rationale for classification **(how the acupuncture points were selected)
**TRADITIONAL ACUPUNCTURE**				

Dong H, Ludicke F et al (2001)	Vasomotor	'Classic manual acupuncture (based on the principles of TCM)'	11	Diagnosis and treatment was given based on the interpretation of symptoms and signs of based on TCM; treatment was individualised.

Huang MI, Nir Y et al (2006)	Vasomotor	Based on 'the principles of TCM'	29	Points were selected from a group that were selected according to TCM *patterns *but the authors mention elsewhere 'based on their ability to target hot flashes'. There was a degree of interpretation, and an individualised approach on the part of the practitioner.

**MEDICAL ACUPUNCTURE**				

Wyon Y, Lindgren R et al (1995)	Vasomotor	'classical acupuncture'	24	No rationale given as to why choosing points for a different syndrome/disease (dysmenorrhoea) except that they didn't know what else to do. As the rationale for treatment is based on matching the mechanism of disease with the mechanism of acupuncture - this group have rationalised that beta-endorphins are the key link.

Hammer M, Frisk J et al (1999)	Vasomotor	-	9	These were male participants given a fixed-point prescription chosen for dysmenorrhoea because the mechanism of vasomotor symptoms is hypothesised to be linked to the production of beta-endorphins.

Cohen SM, Rousseau ME, Carey BL (2003)	Vasomotor	'Within Eastern thought, acupuncture is viewed as a holistic approach grounded in Chinese medical philosophy.'	18	Much TCM language is used in the paper but the points were the same fixed prescription as the other MA vasomotor papers, the rationale for treatment is based on matching the mechanism of disease with the mechanism of acupuncture - this group have rationalised that beta-endorphins are the key link.

Wyon Y, Wigma K et al (2004)	Vasomotor	-	45	As the rationale for treatment is based on matching the mechanism of disease with the mechanism of acupuncture - this group have rationalised that beta-endorphins are the key link and have used the same fixed-point prescription as the other vasomotor studies.

Nedstrand E, Wikma K et al (2005)	Vasomotor	-	38	As the rationale for treatment is based on matching the mechanism of disease with the mechanism of acupuncture - this group have rationalised that beta-endorphins are the key link and have used the same fixed-point prescription as the other vasomotor studies.

**UNCLASSIFIABLE**				

Deng G, Vickers AJ, et al. (2007)	Vasomotor	-	72	The fixed point 'prescription was derived from previous reports and from expert opinion, as found in standard acupuncture textbooks.' It was also changed mid-way through when a change of acupuncturists who wanted to use different points meant the study had to be started again. The is no explanation offered as to why these points might have a certain effect.

Frisk J, Carlhall S et al (2008)	Vasomotor	-	45	Insufficient detail is provided on the acupuncture intervention. Reference is only made to the Wyon Y, Lindgren R et al (1995) study. No other rationale or detail about the treatment is provided.

Harris RE, Tian XM et al (2005)	Fatigue	'The active point formula was chosen based on the points' ability to relieve fibromyalgia symptoms in TCM.'	114	Although the authors describe the study being based on TCM with the knowledge of two acupuncturists, a fixed-point prescription was used according to its effect on symptoms. It is difficult to make further judgement as no rationale was offered as to why these points were chosen or how they might be expected to work.

Kho HG, Eijk RJR et al (1991)	Fatigue	-	29	A fixed-point prescription was used. No information was given regarding rationale for treatment, or mechanism of acupuncture and how it might work in this circumstance.

Malassiotis A, Sylt P, Diggins H. 2006	Fatigue	Point selection was '...based on the Traditional Chinese medicine style, although individualisation was not part of the study.'	47	The points selected are chosen because they have been 'traditionally used for 'energy' over the past 2000 years'. The authors hypothesised why the acupuncture may have worked but this did not drive the fixed-point prescription, which was selected after consultation with two acupuncturists with no rationale offered other than the above or references given.

Martin DP, Sletten CD et al (2006)	Fatigue	-	60	The authors chosen points that 'commonly recur in the acupuncture literature'. No rationale was offered as to why these points might have an effect. A fixed-point prescription was used.

Vickers AJ, Strauss DJ et al (2004)	Fatigue	'Traditional Chinese acupuncture'	37	The authors chose a selection of points that they say are traditionally used to treat fatigue referring to a textbook. There is no index by symptom in this (point-location) textbook thus all points have to be looked up individually and made use of according to TCM theory. A fixed-point prescription is used but is changed during the study with no explanation.

For seven studies [25-31] information is either missing completely or is justified by suggesting that 'experts' chose the points. No clear rationale links the intervention to the outcome, and there is no discussion on any potential mechanisms at play. These studies may imply that acupuncture is a fixed, homogenous intervention and there is no reference to the complex mechanisms at play in the symptoms or what might be changed by the acupuncture. It is thus hard to ascertain what the researchers think might be 'working'. One common problem with these studies is that some suggest by description that they are using a TA model while they are actually using a fixed-point prescription, a common feature of an MA model. Use of a fixed-point prescription invalidates a description of the study as adhering to a TA model. It is, however, possible that authors have described a needle technique using the language of a TA model and omitted to provide a rationale for their point selection. It may be that their approach was founded within a MA model. If so, it is essential to offer a rationale linking point selection to the biomedical mechanism.

Two studies are characterised by the TA model [32,33]. They use defining aspects as described on the left-hand side of Table 1. Although Dong et al [32] also offers a medical rationale, in addition to a TA one, as to the causal link between acupuncture and vasomotor symptoms this does not dictate how the treatment is carried out. Huang et al's [33] study used a 'placebo needle', which involved plastic rings and tape. This procedure was repeated for the 'real' acupuncture group and practitioners complained of it altering their practice.

Five of the studies use the MA model [34-38]; they are all vasomotor studies and are discussed in more depth below.

#### Exploration by Symptom

Examining the studies by symptom provides additional insight into how certain approaches have evolved.

### Vasomotor Symptoms

Five of the nine vasomotor studies are arguably in the MA category and four come from one group of Swedish researchers [35-38]. Their description of a biomedical mechanism as justifying the use of acupuncture and hence the theory underpinning the intervention was rooted in biomedical science. However there are possible problems in characterising these studies due to the fact that they use acu-points for a symptom other than the outcome measure. The rationale for point selection is reported in Wyon et al [35] and similar language is used in the other four papers:

"As there was very sparse experience of acupuncture therapy for climacteric symptoms, the choice of acupuncture points was a modification of points used in previous studies on acupuncture treatment of dysmenorrhoea."

The authors describe the effects of acupuncture in biomedical terms and match these to a possible mechanism underlying the symptom:

"acupuncture could decrease hot flushes by regulating temperature control through increasing beta-endorphin levels and subsequent inhibition of GnRH."

This begs the question of how specific does the understanding of the biomedical mechanism of the symptom have to be to justify an effect and how strong does the link need to be between theory, anticipated mechanism and actual point selection to ensure a robust model. In addition there is the problem of the hypothesized mechanism of hot flushes and Deng et al [25] are upfront about this:

"it has been hypothesized that acupuncture regulates neurotransmitters involved in thermoregulation. Few data currently support this contention."

Of the other four studies, two follow a TA model [32,33] and two are unclassifiable [25,31]. Both Dong et al [32] and Huang et al [33] describe some of the defining aspects for TA displayed in Table 1 but some are assumed; for instance, offering individualised CM treatment is likely to include a differential diagnosis. Deng et al [25] could not be defined as either TA or MA and Cohen et al [34] apply virtually the same fixed-point prescription as the Swedish studies but use some CM language not linked to the point selection.

### Fatigue

None of the five fatigue studies could be classed as either MA or TA [26-30] and all studies had a weak rationale for the chosen treatment. Some studies allude to a more 'traditional' model. Vickers et al [27] describe their selection of acu-points as:

"these points typically are used in Chinese medicine to treat fatigue,"

citing in support a widely used textbook [39] that is more focussed on point location than theory of CM. An examination of the referenced textbook for one of the chosen points (Spleen 9) does not refer to tiredness or fatigue but rather to

*"abdominal distension, cold and pain of the abdomen, cutting pain in the middle of the intestines, no desire to eat*... ". [p194]

In addition there is no explanation as to why the authors modified the points used during the study. The absence or weakness of a rationale for the chosen treatment in all five studies may be partly due to the possibility that no biomedical mechanism associated with fatigue has yet been discovered. It is acknowledged by Vickers et al [27] that fatigue is certainly a multi-factorial problem.

## Discussion

### Does the Framework Help to Identify the Explanatory Model?

The framework aided identification of large gaps in how the interventions were described as used for these particular symptoms; for instance, in the five MA model studies there was no explicit explanation of why the particular acu-points chosen had a particular action. Journal restrictions on word count may have contributed to the limited detail on the nature and rationale for the intervention. But it is also the case that it reflects the particular symptoms focussed on: there is greater clarity on the biomedical theories of pain and less certainty regarding the mechanisms of symptoms. The studies may also have been made to 'fit' a trial design considered ideal for testing simple, single action, fixed interventions such as drugs. It is possible that the theory of the explanatory model is inadequate to explain the link between the practise and the outcome. Studies from the 'unclassifiable' category offer good examples of how trial design shapes practice. More problematically, lack of detail could be due to implicit assumptions about what acupuncture is. Imposition of a RCT model on acupuncture trials may have confounded clarity over model validity to either a TA or MA form; notwithstanding these two models broadly capture the difference between a symptom-based approach compared to a whole person individualised approach and demand a rationale for the mechanism of action.

The framework not only exposes gaps in reporting, but also highlights issues in exploring effectiveness of acupuncture. If a cumulative evidence base is to be developed on the effectiveness of complex interventions such as acupuncture, ensuring both validity and comparability of the intervention is essential. Trials undertaken within a MA model should be compared with another, and those within a TA model one similarly. Comparison of the two models is only warranted when interest lies in the question of the form, 'does a MA or TA model produce better outcomes?'

### Notions of Causality

Different notions of causality may be implicit within the theory of an intervention. In the case of MA, causality is biomedical and linear, whereas in TA the causal logic implied is recursive and non-linear. This adds to the complexity of an intervention. Whatever way the intervention is described, (for instance, 'classical' or 'traditional') it is the rationale that drives the selection of acu-points that exposes the true theoretical basis for treatment.

### What are the Gaps in the Knowledge?

It is evident from this selection of clinical trials that some researchers have rationalised an acupuncture intervention for the use on outcomes other than that being measured based on a hypothesized effect that may link the two. Lack of empirical data may be a reasonable rationale for this; but there is a large body of knowledge available in China, through different branches of science and history that may provide better rationales. Confidence in the model of MA, and in the results of the studies, would be increased if it was possible for authors to explain why certain acu-points, as opposed to others, or even any, have anticipated effects on biomedical mechanisms.

### Implications

Measuring the effectiveness or efficacy of acupuncture is complex, not least because it is difficult to pick apart characteristic and non-specific effects. Studies that clearly define how they do what they do and why they are doing it are more likely to have results that satisfy a range of possible stakeholders than studies that offer an intervention that lacks model validity. It is also important for authors to avoid descriptive short cuts; describing a traditional needle technique cannot be taken as a proxy for the use of CM theory as a basis for a TA model.

Researchers need to provide sufficient information to demonstrate the way in which they have utilised a particular model of acupuncture and as an aid to others evaluating their studies. To assist this process, Table 3 presents a draft tool as a first step to explore and assess model validity in acupuncture interventions. It presents a set of six core questions and associated guiding prompts to ask of a study of acupuncture and may be especially useful where the desired outcomes or symptoms treated have a more complex or unknown mechanism than, for instance, pain. Such a tool requires further exploration, testing and refinement before it can be adopted.

**Table 3:** Exploratory guide for assessing acupuncture model validity (for complex outcomes)

Key Questions	Guiding Prompts
1. What is the underlying *theory *of healing and rationale?	• Derived or based within a TA model?
	• Bio-medically based?
	• Symptom based?
	• Pattern differentiation (based on the approach of looking, listening, asking, touching)?

2. What form does the *diagnosis *of the patient take?	• Symptom based?
	• Pattern differentiation (based on the approach of looking, listening, asking, touching)?
	• Aetiology of the main complaint (detail and depth of exploration)?

3. What is the nature of the *treatment *process?	• Fixed set of acu-points selected according to the main symptom(s)?
	• Is the treatment individualised?
	• Treatment is characterised according to the diagnosis of the whole person using pattern differentiation?

4. How is *treatment modified *at subsequent treatment sessions?	• Treatment modified in response to patient-reported change?
	• Treatment staying constant focussing only on the (main) symptom(s)?

5. What is the implied *mechanism of change *for how the acupuncture might work?	• Biomedical mechanism of the acu-points are linked directly to the symptom?
	• Change is dependent on affecting the balance of Qi, Yin and Yang and other imbalances as defined by the differential diagnosis?

6. What *outcomes *are explored and what is the underlying rationale?	• Resolution of symptoms?
	• Broader benefits on health and well-being?

For the production of robust data, it is not acceptable to use acupuncture in a clinical trial without explicitly describing the theoretical basis of how it is going to work and the rationale for treatment in the published reports [10]. However, it is important to recognise that hypothesised theoretical mechanisms expounded in published trials may be different from the theory-based practice undertaken in real world settings [40].

It is possible that the alien technical language of CM is not seen as appropriate for publication and thus the mechanism of acupuncture according to CM is often not described in detail, but can be assumed from the description of theory. One possible step forward would be for journal editors to agree to publish further, web-based, information in parallel to the trial report.

The framework as developed proved to be a useful aid for identifying and exploring the theory underlying acupuncture intervention trials. It needs further testing with other studies of acupuncture, or non-English language studies, addressing different symptom groups, in order to assess its wider value. This is particularly important given the extensive literature on acupuncture published within the Chinese language [41,42].

## Summary

The aim of this paper was to present the development of a framework and to see if it helped to identify the explanatory model and underlying rationale in published reports on acupuncture trials. It complements the STRICTA guidelines by exploring in greater detail the 'acupuncture rationale'. The insight provided by use of the framework, that is, 'what is the nature of acupuncture as explored within this research study?' is valuable in three main ways. Firstly, at its most simple, it explicitly draws out 'what (form)' and 'how' acupuncture has been undertaken. Secondly, it enables comparison of trials that use a similar approach with one another, rather than putting all acupuncture trials together as if they were all the same (that is, comparing like with like), both in discussions of the implications of a single trial in relation to others and within a systematic review. Thirdly, it may contribute to current debates concerning 'model validity' and what works in acupuncture. Our application of the framework to these selected English language studies suggests that acupuncture trials frequently fail to either apply or report enough detail when using an acupuncture intervention. We would advocate the application of the acupuncture model validity guide to a range of acupuncture trials, along with its further testing and refinement.

## Competing interests

The authors declare that they have no competing interests.

## Authors' contributions

SP conducted the original literature review, drafted the framework and the original manuscript. AFL proposed the realist evaluation method and KT the original review and development of the framework. MG contributed to the whole paper, and specifically to the subject of causality. All authors read, contributed to the re-drafting of the framework and approved the final manuscript.

## Pre-publication history

The pre-publication history for this paper can be accessed here:

http://www.biomedcentral.com/1472-6882/11/22/prepub

## References

[B1] Paterson C Britten N Acupuncture as a complex intervention: a holistic model Journal of Alternative and Complementary Medicine 2004 10 5 791 801 10.1089/acm.2004.10.791 15650468

[B2] MacPherson H Thorpe L Thomas KJ Beyond needling - therapeutic processes in acupuncture care: a qualitative study nested within a low-back pain trial Journal of Alternative and Complementary Medicine 2006 12 9 873 880 10.1089/acm.2006.12.873 17109578

[B3] Paterson C Dieppe P Characteristic and incidental (placebo) effects in complex interventions such as acupuncture BMJ 2005 330 1202 05 10.1136/bmj.330.7501.1202 15905259 PMC558023

[B4] Cheuk DKL Yeung WF Chung KF Wong V Acupuncture for insomnia Cochrane database of systematic reviews 2007 3 Art. No. CD005472 10.1002/14651858.CD005472.pub2 17636800

[B5] Ezzo JM Richardson MA Vickers A Allen C Dibble SL Issell BF Lao L Pearl M Ramirez G Roscoe JA Shen J Shivnan JC Streitberger K Treish I Zhang G Acupuncture-point stimulation for chemotherapy-induced nausea or vomiting The Cochrane Database of Systematic Reviews 2006 4 10.1002/14651858.CD002285.pub2 16625560

[B6] Smith CA Hay PPJ Acupuncture for depression Cochrane database of systematic reviews 2005 2 Art. No. CD004046 10.1002/14651858.CD004046.pub2 15846693

[B7] Birch S Problems with systematic reviews of acupuncture What should we do about these? Clinical Acupuncture and Oriental Medicine 2004 4 105 108 10.1016/S1461-1449(03)00062-8

[B8] MacPherson H White A Cummings M Jobst K Rose K Niemtzow R Standards for reporting controlled trials of acupuncture: the STRICTA recommendations Complementary Therapies in Medicine 2001 9 246 249 10.1054/ctim.2001.0488 12184354

[B9] MacPherson H Altman DG Hammerschlag R Youping L Taixiang W White A Moher D Revised Standards for Reporting Interventions in Clinical Trials of Acupuncture (STRICTA): Extending the CONSORT Statement Plos Med 2010 7 6 e1000261 10.1371/journal.pmed.1000261 20543992 PMC2882429

[B10] Verhoef MF Lewith G Ritenbaugh C Boon H Fleishman S Leis A Complementary and alternative medicine whole systems research: beyond identification of inadequacies of the RCT Complementary Therapies in Medicine 2005 13 206 212 10.1016/j.ctim.2005.05.001 16150375

[B11] Cassidy C Moffet on the similarity of response to "active" and "sham" acupuncture Journal of Alternative and Complementary Medicine 2009 15 3 209 210 (editorial) 10.1089/acm.2009.0070 19292651

[B12] Cassidy CM Model fit validity: seeking a balanced equation Journal of Alternative and Complementary Medicine 2009 15 3 1265 1266 (letter)

[B13] Jonas WB Building an evidence house: challenges and solutions to research in complementary and alternative medicine Forschende Komplementärmedizin/Research in Complementary Medicine 2005 12 3 155 159 10.1159/000085412 15985781

[B14] Mason S Tovey P Long AF Evaluating complementary medicine: methodological challenges of randomised controlled trials BMJ 2002 325 832 10.1136/bmj.325.7368.832 12376448 PMC1124333

[B15] Long AF Outcome measurement in complementary and alternative medicine: unpicking the effects Journal of Alternative and Complementary Medicine 2002 8 6 777 786 10.1089/10755530260511793 12614531

[B16] Paterson C Baarts C Launsø L Verhoef MJ Evaluating complex health interventions: a critical analysis of the 'outcome' concept BMC Complementary and Alternative Medicine 2009 9 18 10.1186/1472-6882-9-18 19538715 PMC2712450

[B17] Craig P Dieppe P MacIntyre S Mitchie S Nazareth I Petticrew M Developing and evaluating complex interventions: the new Medical Research Council guidance BMJ 2008 337 979 983 10.1136/bmj.a1655 PMC2769032 18824488

[B18] Pawson R Greenhalgh R Harvey G Walshe K Realist review - a new method of systematic review designed for complex policy interventions Journal of Health Services Research Policy 2005 10 S1 21 33 10.1258/1355819054308530 16053581

[B19] Pawson R Tilley N Realistic Evaluation 1997 Sage publications

[B20] Hughes JG Goldbard J Fairhurst E Knowles K Exploring acupuncturists' perceptions of treating patients with rheumatoid arthritis Complementary Therapies in Medicine 2007 15 2 101 8 10.1016/j.ctim.2006.09.008 17544860

[B21] Department of Health 2008 30 report to ministers from the department of health steering group on the statutory regulation of practitioners of acupuncture, herbal medicine, traditional Chinese medicine, and other traditional medicine systems practised in the UK

[B22] Kaptchuk TJ Chinese medicine the web that has no weaver 1983 1 Hutchinson Publishing Group 245 254 (in chapter 9)

[B23] Han JS Terenius L Neurochemical basis of acupuncture analgesia Annu Rev Pharmacol Toxicol 1982 22 193 220 10.1146/annurev.pa.22.040182.001205 7044284

[B24] Madsen MV Gotzsche PC Hrobjarrtson A Acupuncture for pain: a systematic review of randomised controlled trials, with acupuncture, placebo acupuncture and no acupuncture groups BMJ 2009 338 a3115 10.1136/bmj.a3115 19174438 PMC2769056

[B25] Deng G Vickers AJ Yeung KS D'Andrea GM Xiao H Heerdt AS Sugarman S Troso-Sandoval T Seidman AD Hudis CA Cassileth BR Randomised Controlled Trial of Acupuncture for treatment of hot flashes in breast cancer patients Journal of Clinical Oncology 2007 25 35 5584 5590 10.1200/JCO.2007.12.0774 18065731

[B26] Kho HG Eijk RJR Kapteijns WMMJ van Egmond J Acupuncture and transcutaeneous stimulation analgesia in comparison with moderate-dose fentanyl anaesthesia in major surgery Anaesthesia 1991 46 2 129 35 10.1111/j.1365-2044.1991.tb09359.x 1908190

[B27] Vickers AJ Strauss DJ Fearon B Cassileth BR Acupuncture for post-chemotherapy fatigue: a phase II study Journal of Clinical Oncology 2004 22 9 1731 5 10.1200/JCO.2004.04.102 15117996

[B28] Harris RE Tian XM Williams DA Tian TX Cupps TR Petzke F Groner KH Biswas P Gracely RH Treatment of fibromyalgia with formula acupuncture: investigation of needle placement, needle stimulation and treatment frequency Journal of Alternative and Complementary Medicine 2005 11 4 663 71 10.1089/acm.2005.11.663 16131290

[B29] Martin DP Sletten CD Williams BA Berger IH Improvement in fibromyalgia symptoms with acupuncture: results of a RCT Mayo Clinic Proceedings 2006 81 6 749 57 10.4065/81.6.749 16770975

[B30] Malassiotis A Sylt P Diggins H The management of cancer-related fatigue after chemotherapy with acupuncture and acupressure: An RCT Complementary Therapies in Medicine 2007 15 4 228 237 10.1016/j.ctim.2006.09.009 18054724

[B31] Frisk J Carlhall S Kallstrom AC Lindh-Anstrand L Malstrom A Hammer M Long-term follow-up of acupuncture and hormone therapy on hot flushes in women with breast cancer: a prospective, randomised, controlled multi-centre trial Climacteric 2008 11 166 174 10.1080/13697130801958709 18365859

[B32] Dong H Ludicke F Comte I Campana A Graff P Bischof P An exploratory pilot study of acupuncture on the quality of life and reproductive hormone secretion in menopausal women JACM 2001 7 6 651 8 10.1089/10755530152755207 11822613

[B33] Huang MI Nir Y Chen B Schyner R Manber R A randomised controlled pilot study of acupuncture for postmenopausal hot flushes: effect on nocturnal hot flashes and sleep quality Fertility and Sterility 2006 86 3 700 710 10.1016/j.fertnstert.2006.02.100 16952511

[B34] Cohen SM Rousseau ME Carey BL Can acupuncture ease the symptoms of the menopause? Holistic Nursing Practice 2003 17 6 295 9 14650571 10.1097/00004650-200311000-00004

[B35] Wyon Y Lindgren R Lundeberg T Hammer M Effects of acupuncture on climacteric vasomotor symptoms, quality of life and urinary excretion of neuropeptides among postmenopausal women Menopause 1995 2 1 3 12 10.1097/00042192-199502010-00002

[B36] Hammar M Frisk J Grimas O Hook M Spetz AC Wyon Y Acupuncture treatment of vasomotor symptoms in men with prostatic carcinoma; a pilot study The Journal of Urology 1999 161 3 853 6 10.1016/S0022-5347(01)61789-0 10022700

[B37] Wyon Y Wigma K Nedstrand E Hammar M A comparison of acupuncture and oral estradiol treatment of vasomotor symptoms in postmenopausal women Climacteric 2004 7 2 153 64 10.1080/13697130410001713814 15497904

[B38] Nedstrand E Wikma K Wyon Y Hammar M Vasomotor symptoms decrease in women with breast cancer randomised to treatment with applied relaxation or electro-acupuncture: a preliminary study Climacteric 2005 8 3 243 250 10.1080/13697130500118050 16390756

[B39] Deadman P Al-Khafaji M Baker K A manual of acupuncture 1998 JCM Publications

[B40] Wayne PM Hammerschlag R Langevin HM Napadow V Park JB Schyner RN Resolving paradoxes in acupuncture research: a roundtable discussion Journal of Alternative and Complementary Medicine 2009 15 9 1039 1044 10.1089/acm.2009.0466 19757981

[B41] Moher D Pham B Lawson ML Klassen TP The inclusion of reports of randomised trials published in languages other than English in systematic reviews Health Technology Assessment 2003 7 1 77 14670218 10.3310/hta7410

[B42] Pilkington K Richardson J Exploring the evidence: the challenges of searching for research on acupuncture Journal of Alternative and Complement Medicine 2004 10 587 90 10.1089/1075553041323795 15253867

